# Approach to the Patient With Primary Aldosteronism: Role of Molecular Imaging

**DOI:** 10.1210/clinem/dgaf396

**Published:** 2025-07-21

**Authors:** Ada E D Teo, Hieu T N Tran, Chin Meng Khoo, Ismail Osman, Aaron Kian-Ti Tong, Roger S Y Foo, Troy H Puar

**Affiliations:** Division of Endocrinology, Department of Medicine, National University Hospital, Singapore 119074, Singapore; Yong Loo Lin School of Medicine, National University of Singapore, Singapore 117597, Singapore; Department of Endocrinology, Changi General Hospital, Singapore 529889, Singapore; Duke-National University of Singapore (NUS) Medical School, Singapore 169857, Singapore; Department of Endocrinology, University Medical Center, Ho Chi Minh City 700000, Vietnam; Division of Endocrinology, Department of Medicine, National University Hospital, Singapore 119074, Singapore; Yong Loo Lin School of Medicine, National University of Singapore, Singapore 117597, Singapore; Department of Endocrinology, Changi General Hospital, Singapore 529889, Singapore; Department of Nuclear Medicine and Molecular Imaging, Singapore General Hospital, Singapore 169854, Singapore; Yong Loo Lin School of Medicine, National University of Singapore, Singapore 117597, Singapore; Cardiovascular Research Institute, National University Health System, Singapore 117599, Singapore; Department of Endocrinology, Changi General Hospital, Singapore 529889, Singapore; Duke-National University of Singapore (NUS) Medical School, Singapore 169857, Singapore

**Keywords:** laparoscopic adrenalectomy, subtyping, functional/nuclear imaging, steroid hybrid hormones, secondary hypertension

## Abstract

A common yet underdiagnosed cause of secondary hypertension, primary aldosteronism (PA) is characterized by excess aldosterone production, causing hypertension with increased risk of cardio-renal-metabolic complications. Accurate and timely localization of the source of aldosterone excess is crucial for management, in the form of curative adrenalectomy for unilateral aldosterone-producing adenoma or medical management for bilateral adrenal hyperplasia. The current diagnostic algorithm involves adrenal vein sampling (AVS) as the current “gold standard” in determining lateralization of aldosterone secretion, but its technical challenges present significant barriers to timely diagnosis and treatment. Recent technological advancements have contributed to the evolution of molecular imaging modalities such as ^11^C-metomidate positron emission tomography-computed tomography (^11^C-MTO PET-CT). Improved molecular imaging modalities hold significant potential to complement existing diagnostic pathways and refine treatment strategies for PA. This review evaluates different case scenarios comparing the utility of AVS with ^11^C-MTO PET-CT, suggesting a practical approach for its interpretation and highlighting the clinical decision-making process.

Primary aldosteronism (PA) is a heterogeneous group of disorders characterized by renin-independent aldosterone overproduction from either 1 (unilateral) or both (bilateral) adrenal glands. With an estimated prevalence ranging from 3.2% to 25% among patients with hypertension across various patient profiles and populations (1-3), it is the most frequent cause of secondary hypertension. This excess aldosterone is linked to significant morbidity and mortality, putting patients with PA at an increased risk of cardio-renal-metabolic complications compared with age- and sex-matched patients with essential hypertension (4). Therefore, early detection with timely diagnosis and appropriate treatment is important. Despite its prevalence and serious health implications, PA remains underrecognized, underdiagnosed, and undertreated, primarily due to the complexity of its diagnostic pathway coupled with poor clinician awareness (5-8). Further underlining the importance of detection is that PA is a potentially curable cause of hypertension.

It is estimated that less than 1% of patients with PA are diagnosed worldwide, with those identified representing cases of florid PA, or the tip of the iceberg (9). Growing evidence suggests that PA exists on a spectrum, ranging from subclinical stages to overt PA, and includes single-focal, multifocal, or diffuse aldosterone-producing areas (9). The use of immunohistochemical staining with specific monoclonal antibodies to aldosterone synthase (CYP11B2) has advanced our understanding of the pathogenesis of PA (10). Immunohistochemistry reveals the exact site of aldosterone excess. With the HISTALDO consensus (11), about 50% to 80% of adrenals removed for unilateral PA may have “classical” unilateral PA, due to either an aldosterone-producing adenoma (APA; defined as ≥1 cm) or aldosterone-producing nodule (APN; defined as <1 cm) (12). These patients with histological findings of a single discrete lesion are more likely to be cured of hypertension and have better biochemical cure rates in the long term (13), compared to those with “nonclassical” unilateral PA, such as aldosterone-producing micronodule (APM) or adrenal hyperplasia.

## Subtyping of PA

Although it is increasingly recognized that PA exists along a spectrum, PA remains subdivided dichotomously into either unilateral or bilateral PA for the purpose of clinical decision-making regarding treatment (14). While patients with bilateral PA require lifelong management with mineralocorticoid receptor antagonists, those with unilateral PA can be cured through laparoscopic unilateral adrenalectomy. Computed tomography (CT) of the adrenal glands may identify APAs, but it may miss smaller APNs (defined as <1 cm) (15, 16). Additionally, CT scans cannot differentiate between functioning and nonfunctioning adrenal tumors. Instead, adrenal vein sampling (AVS) provides the required functional information: under fluoroscopic guidance, blood samples are taken from both adrenal veins, and a higher aldosterone level in 1 adrenal vein compared to the contralateral side is consistent with unilateral PA. The current Endocrine Society guidelines recommend AVS for subtyping and to lateralize PA in almost all patients (14). The main limitation of AVS is that it is a technically demanding procedure with cannulation of the right adrenal vein particularly difficult. Failure to cannulate either adrenal vein may occur in 20% to 50% of cases, and this leads to an indeterminate result (17). Despite being the reference standard for subtyping PA, AVS remains a bottleneck in patient management. As such, there is increasing interest in identifying alternative modalities for subtyping PA that are less invasive.

## Molecular Imaging as an Alternative Subtyping Test

Molecular imaging has been widely used for the localization of tumors in endocrinology, such as technetium for parathyroid adenomas (18), and ^68^Ga-labeled somatostatin analogues for pheochromocytomas and paragangliomas (19). In the field of PA, radiolabeled cholesterol 6β-^131^Iodomethyl-19-norcholesterol (NP-59) has been used for several decades. While it has been shown to identify unilateral PA, lesions smaller than 1.5 cm may not be visible due to the spatial resolution limit of NP-59 single-photon emission CT/CT planar imaging, thereby limiting its utility in subtype differentiation (20).

Metomidate (MTO) is a potent inhibitor of both CYP11B1 (11β-hydroxylase), which is involved in the last step of cortisol synthesis, and CYP11B2 (aldosterone synthase), which is the main regulator of aldosterone synthesis. In 2000, MTO was successfully ^11^C-labeled as a positron emission tomography (PET) radiotracer (21). To increase the selectivity of ^11^C-metomidate (^11^C-MTO) for CYP11B2, patients are prescribed dexamethasone pre-scan. This reduces CYP11B1 expression in the adrenal glands and allows ^11^C-MTO to be a radioligand specific for detecting APA (22). Coupled with high-resolution CT, ^11^C-MTO PET-CT can detect functional APA less than 1 cm in size and has been shown to offer a noninvasive alternative to AVS in identifying unilateral surgically curable PA (22, 23).

In this review, we discuss selected and illustrative cases from our prospective studies (NCT03990701 and NCT06100367), in which both current gold-standard AVS and molecular imaging ^11^C-MTO PET-CT scans have been used, and the clinical decisions reached in each scenario. The protocols for diagnosis of PA, AVS, and ^11^C-MTO PET-CT have been previously described (22).

## Clinical Cases

### Clinical Case 1

**Figure 1.**
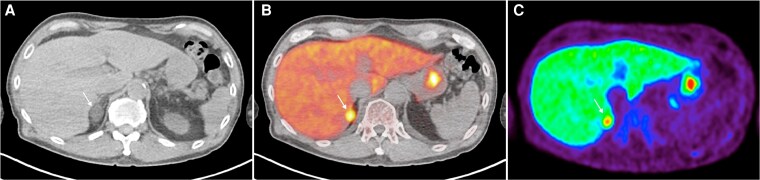
^11^C-MTO PET-CT for case 1—right adrenal nodule (white arrow) seen on CT imaging (A) with a high SUVmax over the right adenoma (32.7) compared to the left adrenal gland (18.7) on the fused PET-CT imaging (B) and PET imaging (C). The SUVmax ratio of right adrenal nodule-to-contralateral gland was 1.75 (greater than cutoff of 1.25 to define lateralization). Abbreviations: ^11^C-MTO PET-CT, ^11^C-metomidate positron emission tomography-computed tomography; SUVmax, maximum standardized uptake value.

Patient 1 is a 65-year-old male with a 20-year history of hypertension with spontaneous hypokalemia, with serum potassium (K+) 2.9 mmol/L. His blood pressure (BP) was 130/80 mmHg on 3 agents: amlodipine 10 mg daily, valsartan 160 mg daily, and doxazosin 8 mg daily. Plasma aldosterone concentration (PAC) was 21.8 ng/dL and plasma renin activity (PRA) was <0.4 ng/mL/hr, with an aldosterone-renin ratio (ARR) of 54.5 ng/dL per ng/mL/hr. This was consistent with a diagnosis of PA, with suppressed renin (despite being on an angiotensin-receptor blocker, which may elevate renin) (24). His cortisol was suppressed (34 nmol/L) after a 1-mg overnight dexamethasone suppression test (ONDST). Adrenal CT imaging revealed a 1.7 × 2 cm homogenous nodule on the lateral limb of the right adrenal gland. On AVS, there was lateralization to the right (aldosterone-cortisol ratio 5.6 times higher on the right compared to the left). Similarly, ^11^C-MTO PET-CT detected a right-sided nodule with intense uptake, maximum standardized uptake value (SUVmax) 32.7, while the left adrenal gland SUVmax was 18.7, yielding an SUVmax ratio of 1.75 (greater than 1.25 cutoff) (Fig. 1). After right adrenalectomy, a 2-cm adenoma was identified on histopathology. Postoperatively, the patient achieved complete biochemical normalization: PAC <3 ng/dL, PRA <0.4 ng/mL/hr, and normokalaemia. His BP improved to 120/77 mmHg on 2 agents: amlodipine 5 mg daily and valsartan 80 mg daily.

#### Learning points (concordant lateralization on CT, AVS, and ^11^C-MTO PET-CT)

- On ^11^C-MTO PET-CT, there is clear lateralization if the highest SUVmax uptake is localized in a visible nodule on CT and the uptake is 1.25 times more compared with the SUVmax of the contralateral gland (22, 23).
- When ^11^C-MTO PET-CT results meet clear lateralization criteria, adrenalectomy can be considered.
- The most common scenario in patients with unilateral PA is concordant lateralization seen on both molecular imaging (^11^C-MTO PET-CT) and AVS. Where available, the noninvasive ^11^C-MTO PET-CT could obviate the need for an invasive AVS and would be an ideal first-line subtype test.

### Clinical Case 2

**Figure 2.**
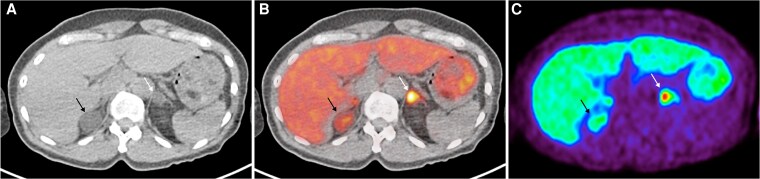
^11^C-MTO PET-CT for case 2—bilateral adrenal nodules seen on CT imaging (A) with a high SUVmax over the left adenoma (64.4) (white arrow) compared to the right adrenal gland (51.1) (black arrow) on the fused PET-CT imaging (B) and PET imaging (C). The SUVmax ratio of left adrenal nodule-to-contralateral gland was 1.26 (greater than cutoff of 1.25 to define lateralization). Abbreviations: ^11^C-MTO PET-CT, ^11^C-metomidate positron emission tomography-computed tomography; SUVmax, maximum standardized uptake value.

Patient 2 is a 63-year-old female with a 23-year history of hypertension. Her BP was 133/85 mmHg on amlodipine 10 mg daily. Her ARR was 22.2 ng/dL/ng/mL/hr, and PAC post salt-loading test (SLT) was 25 ng/dL. Her cortisol level was suppressed to 42 nmol/L after 1 mg ONDST. On CT imaging, there were bilateral adrenal nodules, with a right adrenal adenoma 3.0 × 2.3 cm and a left adrenal adenoma 1.6 × 1.4 cm. AVS revealed clear lateralization to the smaller left-sided nodule with a lateralization index of 25.2. On ^11^C-MTO PET-CT, there was intense uptake on the smaller left nodule, with a corresponding SUVmax ratio 1.26 (greater than 1.25 cutoff) (Fig. 2). She underwent left adrenalectomy and achieved complete biochemical cure: ARR 1.5 ng/dL per ng/mL/hr and normalized K+. Her BP improved to 117/82 mmHg on amlodipine 10 mg daily.

#### Learning points (CT bilateral; AVS and ^11^C-MTO PET-CT concordant lateralization)

- Unlike CT imaging, which cannot differentiate functional from nonfunctional adenomas, ^11^C-MTO PET-CT scan can selectively identify functional APA.
- In this patient with bilateral adrenal nodules, only the smaller left APA was tracer-avid, while the right nonfunctional lesion did not take up significant tracer.
- In this case, both functional biochemistry (AVS) and molecular imaging (^11^C-MTO PET-CT) had concordant lateralization to the left.
- Previous studies have shown that ^11^C-MTO uptake correlates well with CYP11B2 expression but not CYP11B1 expression (23).

### Clinical Case 3

**Figure 3.**
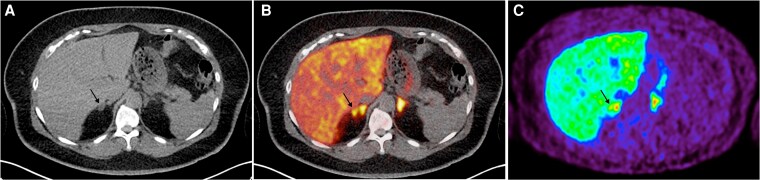
^11^C-MTO PET-CT for case 3—right adrenal nodule seen on CT imaging (A) with low SUVmax over the right medial limb nodule (25.5) compared to the left adrenal gland (28.1) on the fused PET-CT imaging (B) and PET imaging (C). The SUVmax ratio of right adrenal nodule-to-contralateral gland was 0.91 (less than cutoff of 1.25 to define lateralization). Abbreviations: ^11^C-MTO PET-CT, ^11^C-metomidate positron emission tomography-computed tomography; SUVmax, maximum standardized uptake value.

Patient 3 is a 45-year-old female who presented with left ataxic hemiparesis secondary to right capsular infarct, with a 10-year history of resistant hypertension on 3 agents: terazosin 6 mg daily, irbesartan 300 mg daily, and nifedipine 60 mg twice daily. She was noted to have hypokalemia, with serum K+ 2.5 mmol/L. Her screening ARR was 20.0 ng/dL per ng/mL/hr, and PAC level remained elevated at 12 ng/dL post-SLT. Her cortisol was under 50 nmol/L post-1 mg ONDST. Adrenal CT imaging revealed a 1.3 cm hypodense nodule only in the lateral limb of the right adrenal gland. On AVS, the left aldosterone-cortisol ratio was 2.50 and the right was 2.33, with no lateralization. On ^11^C-MTO PET-CT imaging, the right adrenal gland SUVmax was 25.5 (but without uptake over the visible nodule), while the left adrenal gland SUVmax was 28.1 **(**Fig. 3). Hence, this patient was diagnosed as having bilateral PA and started on spironolactone. She is currently on spironolactone 75 mg twice daily, along with terazosin, irbesartan, and nifedipine, with an improved BP and normokalemia (K+ 4.7 mmol/L).

#### Learning points (CT right nodule; AVS and ^11^C-MTO PET-CT no lateralization)

- In patients with bilateral adrenal disease, there is a lack of lateralization with either AVS or ^11^C-MTO PET-CT.
- Although this patient had a visible adenoma on CT, this was not tracer-avid, and there was similar ^11^C-MTO uptake in both adrenal glands.

### Clinical Case 4

**Figure 4.**
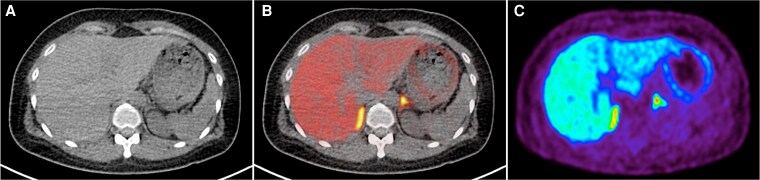
^11^C-MTO PET-CT for case 4—no adrenal lesion seen on CT imaging (A). AVS showed clear left lateralization, but MTO detected a left adrenal nodule with only SUVmax 45.2, compared to SUVmax of 39.5 over the right adrenal body on the fused PET-CT imaging (B) and PET imaging (C). The SUVmax ratio of left adrenal nodule-to-contralateral gland was 1.14 (less than cutoff of 1.25 to define lateralization). Abbreviations: ^11^C-MTO PET-CT, ^11^C-metomidate positron emission tomography-computed tomography; AVS, adrenal vein sampling; SUVmax, maximum standardized uptake value.

Patient 4 is a 37-year-old female with hypertension for 7 years; BP was 140/60 mmHg on amlodipine 10 mg daily. Her ARR was 65.5 ng/dL per ng/mL/hr, and PAC post-SLT was 11.5 ng/dL. Adrenal CT imaging showed no obvious adrenal lesion. On AVS, there was lateralization to the left adrenal, with a lateralization ratio of 5.3 (greater than 4). On ^11^C-MTO PET-CT imaging, there was higher uptake in the left adrenal gland compared to the right, with an SUVmax uptake ratio of 1.14 (below 1.25 cutoff) (Fig. 4). In view of the AVS results, the patient underwent left adrenalectomy. Eight months postsurgery, her PAC was 4.9 ng/dL, PRA 0.9 ng/mL/hr, and BP 116/78 mmHg. However, 2 years after surgery, her PAC had risen to 9.6 ng/dL, PRA 0.6 ng/dL, and BP 135/90 mmHg. She should have monitoring for possible recurrence and consideration of medical therapy with aldosterone antagonists if required.

#### Learning points (CT no nodule; AVS lateralization but ^11^C-MTO PET-CT uptake not reaching threshold)

- Some patients with unilateral PA may not have evident localization on ^11^C-MTO PET-CT but demonstrate lateralization on AVS. They can still be offered unilateral adrenalectomy.
- In about 10% to 20% of patients, there are no APA or APN, and APMs are the source of aldosterone excess (25). In these patients, AVS may identify asymmetrical bilateral PA (26).
- Regardless of the subtype modality used, there is a higher likelihood of recurrence of PA in “nonclassical” unilateral PA (no APA or APN on histopathology). As seen in this case, these patients should be followed up closely for any recurrence (27).

### Clinical Case 5

**Figure 5.**
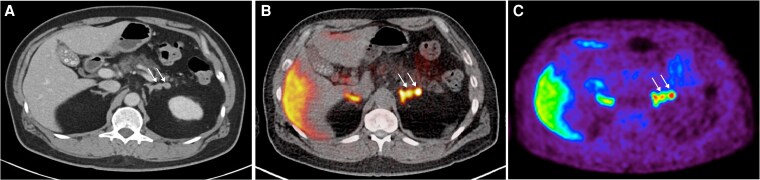
^11^C-MTO PET-CT for case 5—3 small left adrenal nodules detected on CT imaging (A). MTO showed 2 nodules over left lateral limb with high uptake of SUVmax 54.7 (lateral most nodule 1.0 cm) and SUVmax 33.4 (medial most nodule 0.6 cm), compared to the right adrenal gland (34.4) on the fused PET-CT imaging (B) and PET imaging (C). The SUVmax ratio of left adrenal nodules to contralateral gland was 1.59 and 0.97, respectively (compared to cutoff of 1.25 to define lateralization). Abbreviations: ^11^C-MTO PET-CT, 11C-metomidate positron emission tomography-computed tomography; SUVmax, maximum standardized uptake value.

Patient 5 is a 48-year-old male with a 10-year history of hypertension with episodes of severe hypokalemia (K+ 1.9 mmol/L). He was treated with amlodipine and hydralazine. His ARR was 26.7 ng/dL per ng/mL/hr, and post-SLT PAC was 6.1 ng/dL. CT imaging revealed 3 small nodules in the lateral limb of the left adrenal gland (lateral-most 1.0 cm, middle 0.8 cm, medial-most 0.6 cm). On AVS, there was no lateralization, with a lateralization index of 2.3 toward the left (below 4 cutoff). However, on ^11^C-MTO PET-CT, there were 2 distinct tracer-avid nodules in the left lateral limb with SUVmax 54.7 (lateral-most 1.0 cm nodule) and SUVmax 33.4 (medial-most 0.6 cm nodule), compared to the contralateral adrenal gland SUVmax of 34.4, with a SUVmax ratio of 1.59 (greater than 1.25 cutoff) (Fig. 5). This patient underwent left adrenalectomy with complete biochemical and clinical cure of hypertension postsurgery: normalized ARR 1.5 ng/dL per ng/mL/hr and BP 124/88 mmHg on amlodipine. Evaluation of subsequent histology revealed 4 APNs (<1 cm), with predominantly lipid-poor zona glomerulosa cells.

#### Learning points (CT multiple left nodules; AVS no lateralization but ^11^C-MTO PET-CT lateralization)

- Some patients with unilateral PA may demonstrate lateralization on ^11^C-MTO PET-CT but not on AVS, and these patients may also be offered adrenalectomy.
- In patients with failed cannulation on AVS, ^11^C-MTO PET-CT offers another opportunity to identify unilateral disease.
- ^11^C-MTO PET-CT demonstrates good spatial resolution and can identify functional APN as small as 6 mm.
- As CYP11B2 expression is negatively correlated with tumor size (28), due to its high affinity for CYP11B2, ^11^C-MTO PET-CT has been reported to be particularly effective in identifying smaller functional APA (22, 29).

## AVS: Utility and Limitations

To accurately distinguish between unilateral and bilateral PA, AVS has been the ideal subtype test for several decades. Young and colleagues found that relying solely on CT imaging would have led to 21.7% of patients being incorrectly excluded from adrenalectomy and 24.7% undergoing unnecessary surgery (15), while Kempers et al reported a 37.8% discrepancy rate between AVS and CT results (16), which could have resulted in inappropriate treatment decisions. Due to the challenges in performing AVS, some patients may undergo adrenalectomy based on CT findings when AVS is either unavailable or unsuccessful. A randomized controlled trial comparing outcomes of CT- vs AVS-guided adrenalectomy found no significant differences in BP between the groups but a trend toward lower biochemical remission in the CT group (30). In a larger retrospective study, AVS-guided adrenalectomy was shown to have superior biochemical success and similar clinical success compared to CT-guided adrenalectomy (31). These findings highlight the critical role of AVS in guiding appropriate surgical interventions, ensuring accurate lateralization of aldosterone hypersecretion and ultimately ensuring good patient outcomes.

The main limitation with AVS is that it is an invasive and technically challenging procedure, and bilateral successful cannulation is ∼80% even in highly specialized centers (17). The use of intraprocedure ACTH stimulation improves successful AVS by maximizing adrenal cortisol secretion and increasing the adrenal-to-peripheral cortisol gradient, which can be low during baseline sampling (32). However, ACTH stimulation may reduce the lateralization index, resulting in some patients with unilateral PA falsely classified as having bilateral PA (33-35). As AVS protocols and biochemical thresholds to determine unilateral disease vary between centers, this adds to the complexity in determining lateralization. Results of AVS may also be confounded by cortisol co-secreting APAs, venous anomalies, or placement of catheters, resulting in inadvertent super-selective sampling (36-38).

Almost all patients with lateralization on AVS achieved biochemical cure after unilateral adrenalectomy. However, 3% to 6% of patients may have persistent hyperaldosteronism (39). One reason is that asymmetrical aldosterone overproduction occurs in bilateral PA (40). Since AVS compares the aldosterone production from both adrenal glands, patients with bilateral asymmetrical aldosterone production may show lateralization on AVS. Even patients with marked lateralization may still have biochemical persistence or recurrent post-adrenalectomy (26). Factors associated with a higher likelihood of biochemical persistence post-adrenalectomy include patients of Black/African ancestry, lateralization on AVS but CT shows bilateral normal (or abnormal) adrenal morphology, and AVS lateralization only without ACTH (and absent lateralization with ACTH) (26). Hence, the presence of a visible nodule on CT imaging may portend better postsurgical outcomes.

## ^11^C-MTO PET-CT: Utility and Limitations

Clinical cases 1, 2, and 5 illustrate that ^11^C-MTO can be a highly effective radiotracer for identifying functional APAs. In clinical cases 3 and 4, nonfunctional adenomas had low affinity for ^11^C-MTO and were PET-negative, demonstrating the specificity of ^11^C-MTO for CYP11B2 activity (22, 23, 29). Due to the 95% homology between the CYP11B1 and CYP11B2 genes, ^11^C-MTO exhibits low selectivity for CYP11B2 over CYP11B1. In a previous study by Burton and colleagues (29), the use of 0.5 mg dexamethasone given every 6 hours for 72 hours before the scan reduced the uptake of ^11^C-MTO in the normal adrenal gland, thus increasing the lateralization ratio in patients with unilateral PA. This is crucial when using ^11^C-MTO PET-CT for the subtyping of PA. In this study, a tumor SUVmax to normal adrenal gland ratio of 1.25 and greater was shown to have a sensitivity of 76% and specificity of 87%, compared to AVS, for the diagnosis of unilateral PA. The importance of dexamethasone premedication was substantiated by another study, where ^11^C-MTO PET-CT fared poorly when performed without dexamethasone: Soinio and colleagues reported that when ^11^C-MTO PET-CT was compared to AVS, the area under receiver operating characteristics curve was 0.507 to predict unilateral PA, with a sensitivity of 55% and specificity of 44% in the absence of dexamethasone premedication (25).

In 2019, ^11^C-MTO PET-CT was first reported to be useful in diagnosing patients with unilateral PA when AVS failed or was indeterminate (41). In 2022, in a prospective clinical trial comparing ^11^C-MTO PET-CT (with dexamethasone) vs AVS in 25 PA patients (PA_CURE) (22), all 25 patients had successful AVS and also underwent ^11^C-MTO PET-CT. Twenty patients underwent surgery, with all experiencing biochemical cure after 6 months as defined by the primary aldosteronism surgical outcome (PASO) criteria. Of the 20 patients, 12 (60%) were identified with unilateral PA on both modalities, 4 (20%) on ^11^C-MTO PET-CT only, and 3 (15%) on AVS only. The final patient had lateralization on the second AVS, despite having an initially successful first AVS. This was likely due to the placement of the catheter in an adrenal tributary, which did not drain the APA, highlighting a limitation of AVS. This study illustrated the possibility of ^11^C-MTO PET-CT identifying patients with unilateral PA who were missed on AVS.

In 2023, in the largest study to date, the MATCH study (23) demonstrated that ^11^C-MTO PET-CT was noninferior to AVS. In this UK study, 86 of 128 patients were diagnosed with unilateral disease, of which 78 underwent surgery, with 77 of 78 patients achieving one of the prespecified composite outcomes: improvement or cure in biochemical or clinical PASO outcomes. Of the 86 patients, 39 were identified with unilateral PA on both modalities, 28 on ^11^C-MTO PET-CT only, and 19 on AVS only. In patients with unilateral PA, the most common scenario is concordance between ^11^C-MTO PET-CT and AVS: unilateral PA to the same adrenal gland (cases 1 and 2) or bilateral PA (case 3). Hence, in most cases, the availability of ^11^C-MTO PET-CT may potentially obviate the requirement to perform AVS if clear lateralization is seen.

One reason for this apparent discrepancy between AVS and ^11^C-MTO PET-CT is the use of strict cutoffs to determine unilateral PA: an AVS lateralization index of 4 and above and an SUVmax ratio on ^11^C-MTO PET-CT of 1.25 and above. While PA is increasingly recognized as a spectrum, its diagnosis and management still rely on arbitrary cutoffs, highlighting the inherent issue with the dichotomous approach in classifying PA. Just as AVS lateralization indices use cutoffs of 2 to 4, depending on whether ACTH stimulation is included, ^11^C-MTO PET-CT also applies predefined thresholds to categorize patients as having unilateral or bilateral PA, reinforcing the limitations of rigid dichotomization in clinical decision-making. Since AVS only provides functional information, patients with lateralization on AVS could have nonclassical unilateral PA (eg, hyperaldosteronism from APN). Conversely, since ^11^C-MTO PET-CT integrates both structural and functional information, ^11^C-MTO PET-CT may be better at identifying classical unilateral PA (discrete APA or APN) in patients who have higher rates of biochemical and clinical success, compared to those with multiple functional nodules or hyperplasia (termed “nonclassical”) (13, 40).

Molecular imaging using ^11^C-MTO offers several other advantages over AVS. First, molecular imaging is noninvasive and can be safely performed in patients prescribed antiplatelets/anticoagulants or those with bleeding diathesis. Second, molecular imaging is not dependent on highly skilled proceduralists, such as in the case of AVS. It can be performed in an ambulatory setting and does not require angiography suites or hospitalization beds. Third, molecular imaging is not affected by venous anomalies, which may either affect successful cannulation or lead to erroneous interpretation (42-44). Finally, by localization to the functional APA or APN, patients may potentially be offered directed therapy toward the nodule [eg, radiofrequency ablation (RFA)]. This is not recommended with AVS as the adrenal gland with abnormally high aldosterone production is identified and the adrenal gland has to be removed in its entirety.

However, ^11^C-MTO PET-CT has several important limitations. First, ^11^C-MTO has a short 20-minute half-life, thereby requiring synthesis by an on-site cyclotron, and cannot be transported to distant sites. Hence, it is unlikely to be scaled to centers worldwide. Second, ^11^C-MTO demonstrates a fair amount of nonspecific liver uptake, which may make uptake over right-sided adrenal lesions more challenging to distinguish, especially in individuals with less adipose tissue separating the liver from the right adrenal gland. Third, due to the low selectivity of ^11^C-MTO for CYP11B2 compared with CYP11B1, pretreatment with dexamethasone to suppress CYP11B1 in patients undergoing ^11^C-MTO PET-CT is required. Dexamethasone may lower aldosterone levels even in patients with PA (45). However, findings from the aforementioned studies suggest that dexamethasone premedication still allows accurate localization of APA in a majority of cases. There are currently few data on cortisol co-secreting APA or studies of patients with co-existing cortisol-producing adenomas, where interpretation of both AVS and ^11^C-MTO PET-CT may be difficult, even when imaging after dexamethasone suppression. Finally, ^11^C-MTO PET-CT imaging was performed by an experienced nuclear medicine physician and discussed in multidisciplinary meetings in these centers. With wide applications of molecular imaging currently in endocrinology, we expect that the emerging application of molecular imaging for subtyping PA should not be a major hurdle.

## Other Molecular Imaging Markers in the Horizon

### Para-chloro-2-[^18^F]fluoroethyl-etomidate

**Figure 6.**
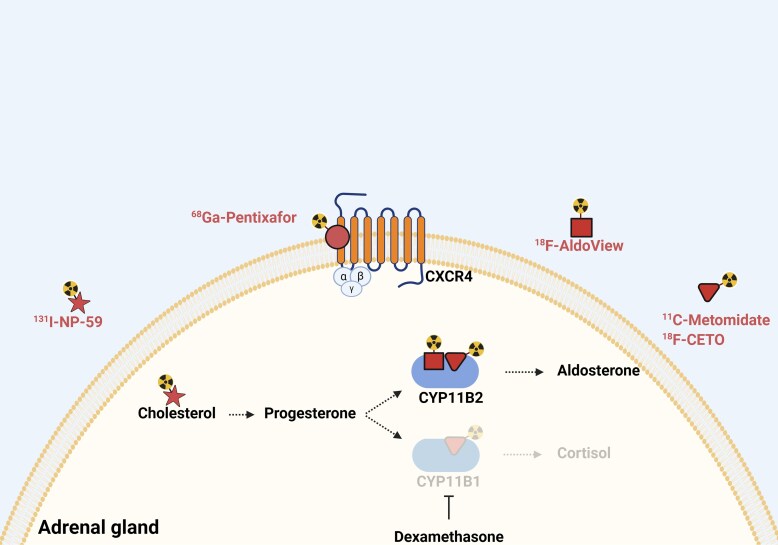
Main radiotracers developed over the past decade and their binding locations. Created with BioRender.com.

Besides optimizing data and image analysis, current efforts also have moved toward exploring alternative tracers with longer half-lives. Figure 6 illustrates the main radiotracers developed over the past decade for imaging APA. The development of more stable isotopes, such as para-chloro-2-[^18^F]fluoroethyl-etomidate (^18^F-CETO), is likely to significantly increase accessibility to molecular imaging. The fluorine-18-labeled MTO analog ^18^F-CETO has a high binding specificity to the adrenal cortex and low nonspecific uptake in the liver compared to ^11^C-MTO (46). With a half-life of 2 hours and a high lesion-to-background contrast ratio, ^18^F-CETO PET-CT simplifies workflow in the clinical setting and may provide better visualization of smaller lesions compared to ^11^C-MTO PET-CT (47). In a phase I/IIa trial, ^18^F-CETO PET-CT demonstrated high selectivity for the adrenal glands with low uptake in other organs (48). However, similar to ^11^C-MTO PET-CT, dexamethasone premedication is required to improve visualization of APA. It is currently undergoing clinical trial evaluation for its use in PA diagnosis (NCT04529018).

### ^18^F-AldoView

Other ^18^F-radiotracers developed recently include the benzimidazole derivative, an aldosterone synthase inhibitor labeled with fluorine-18, to generate a highly CYP11B2-selective radiotracer known as ^18^F-AldoView (49). In preclinical studies in mice, this PET tracer showed a favorable pharmacokinetic profile that included rapid distribution and clearance of the tracer (50). In addition, binding studies in tissue sections from surgically resected adrenals provided evidence for highly- selective binding to CYP11B2 (50). As such, ^18^F-AldoView may be advantageous as it does not require dexamethasone pretreatment. Currently, ^18^F-AldoView is awaiting first-in-human studies.

### ^68^Ga-Pentixafor

In terms of differentially expressed cell surface receptors, the CXC chemokine receptor type 4 (CXCR4) has been reported to be upregulated in APAs with almost negligible expression in nonfunctional adenomas based on immunohistochemistry (51). In 2020, the cyclic pentapeptide Pentixafor was developed to bind CXCR4 with high affinity, with adequate distribution profile and rapid renal excretion (52). In a study of 9 patients with PA, there was focal ^68^Ga-Pentixafor uptake in the culprit adrenal nodule with SUVmax values ranging between 4.7 and 18.3 (mean 10.7), while the mean SUVmax value was 3.3 in the normal adrenal gland (51). In a larger study of 100 patients with PA, the concordance rate of ^68^Ga-Pentixafor PET-CT and AVS was 100% in patients with a unilateral APA 1 cm or greater on CT. However, in patients without a typical unilateral nodule on CT, ^68^Ga-Pentixafor was only able to identify about half of these patients, indicating that the spatial resolution of ^68^Ga-Pentixafor may not be adequate to detect smaller lesions below 1 cm (53). In addition, immunohistochemistry revealed low CXCR4 staining in 29% of APAs (51), suggesting that false-negative imaging may occur with ^68^Ga-Pentixafor. More recent prospective studies have also supported the ability of ^68^Ga-Pentixafor PET-CT to accurately localize unilateral APAs (54, 55). Currently, image acquisition time intervals and thresholds to define lateralization using ^68^Ga-Pentixafor PET-CT differ between studies, and the optimal protocol needs to be further established.

## Future Directions

**Figure 7.**
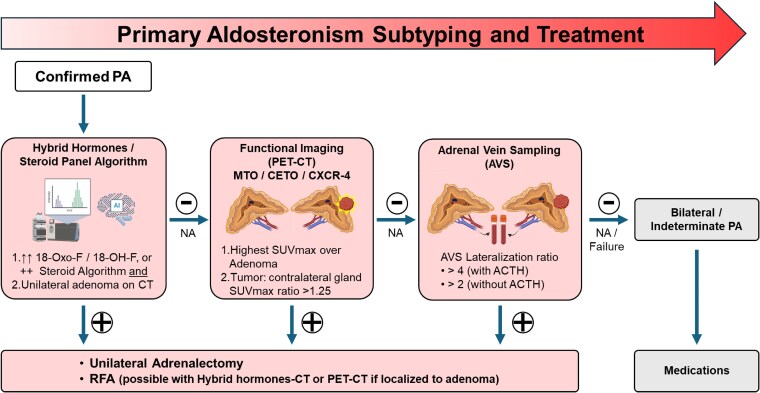
Proposed future algorithm for subtyping PA. Current data suggests that each subtype test is highly specific. Hence, this algorithm recommends that they can be used sequentially, moving from the least to the most invasive: hybrid hormone/steroid panel algorithm, molecular imaging, and adrenal vein sampling. *Clear lateralization for ^11^C-MTO PET-CT uses an SUVmax ratio of greater than 1.25; thresholds with other radiotracers would differ. Abbreviations: ^11^C-MTO PET-CT, ^11^C-metomidate positron emission tomography-computed tomography; AVS, adrenal vein sampling; NA, not available; PA, primary aldosteronism; SUVmax, maximum standardized uptake value.

While shown to be highly accurate, molecular imaging and AVS are not available in some countries. A previous study reported that 0.5 billion of the world's population in Southeast Asia alone had no access to subtype tests (either PET-CT imaging or AVS) (56). Future scalable subtype tests would be crucial to address this gap in diagnosing a highly prevalent condition like PA. Of particular interest is the use of serum biomarkers to identify unilateral PA (Fig. 7). Hybrid hormones such as 18-hydroxycortisol and 18-oxocortisol (57, 58), are high in unilateral PA, particularly APAs that harbor a somatic mutation in the *KCNJ5* gene (59). This encodes the inwardly rectifying potassium channel, whereby its mutation results in membrane depolarization and autonomous aldosterone secretion (60).

*KCNJ5*-mutant APAs are more common among Asians (60-70%) than Europeans (30-40%) (61-63). While patients with *KCNJ5*-mutant APA often have severe hypertension, they are also the most likely to be cured of hypertension postsurgery (64). *KCNJ5*-mutant APAs are often large (>1 cm) and visible on CT imaging. Hence, it is possible that, in the future, elevated hybrid hormones in the presence of a CT-visible adenoma may be adequate for patients to be recommended adrenalectomy. A more in-depth review into the utility of hybrid hormones has been published previously (65). Furthermore, compared to measuring only these 2 hybrid hormones, complete steroid profiling of adrenal hormones coupled with machine-learning artificial intelligence has been shown to offer superior diagnostic accuracy for diagnosis and subtyping of PA (66, 67). Furthermore, using a complete steroid profile and probabilistic scoring approach may be less affected by the use of BP medications and offer better accuracy (68).

Compared to unilateral laparoscopic adrenalectomy, RFA is less invasive, has a shorter operating time and shorter hospital stay, and can be performed under sedation with a shorter recovery time (69). Previous studies have shown that the long-term biochemical success rates with RFA are about 60% to 80% (70-72). This is currently lower than that with laparoscopic adrenalectomy. However, RFA was directed based on AVS-guided lateralization. With the ability of molecular imaging to localize the source of hyperaldosteronism, outcomes with RFA may improve. Since RFA only ablates the hyperfunctioning adrenal tumor, this may preserve surrounding normal adrenal tissue and open up the possibility of treating patients with bilateral APA. There are also newer, less invasive techniques for RFA, including the transvenous approach (73) or transgastric approach. The recent FABULAS study (74) demonstrated the feasibility and safety of endoscopic, ultrasound-guided RFA for left-sided APAs, with 75% achieving biochemical success and 43% showing clinical improvement or cure of hypertension at 3-months postablation, while maintaining a strong safety profile with no major complications.

## Conclusion

This advent in molecular imaging is timely given the recent recognition that PA is the most common, potentially curable, cause of secondary hypertension. Contributing to a significant fraction of the adult hypertensive burden, newer molecular imaging modalities and potential biomarker-directed options offer a welcome addition to the existing invasive option of AVS for subtyping of PA. This would hopefully allow more patients to progress to the end of the diagnostic pathway, increasing the number of patients who can undergo potentially curative surgery and be spared lifelong medical therapy.

## Data Availability

Data sharing is not applicable to this article as no datasets were generated or analyzed during the current study.
